# Clinical, radiological, and laboratory predictors of a positive urine lipoarabinomannan test in sputum-scarce and sputum-negative patients with HIV-associated tuberculosis in two Johannesburg hospitals

**DOI:** 10.4102/sajhivmed.v22i1.1234

**Published:** 2021-07-08

**Authors:** Lior Chernick, Ismail S. Kalla, Michelle Venter

**Affiliations:** 1 Department of Internal Medicine, Faculty of Health Sciences, University of the Witwatersrand, Johannesburg, South Africa; 2 Department of Internal Medicine, Chris Hani Baragwanath Academic Hospital, Johannesburg, South Africa; 3 Division of Pulmonology, Department of Internal Medicine, Charlotte Maxeke Johannesburg Academic Hospital, Johannesburg, South Africa; 4 Division of Infectious Diseases, Department of Internal Medicine, University of the Witwatersrand, Johannesburg, South Africa

**Keywords:** HIV, TB, lipoarabinomannan, sputum negative, sputum scarce

## Abstract

**Background:**

Tuberculosis (TB) is a major cause of mortality in persons living with HIV (PLWH). Sputum-based diagnosis of TB in patients with low CD4 counts is hampered by paucibacillary disease and consequent sputum scarcity or negative sputum results. Urine lipoarabinomannan (LAM) has shown promise in the point-of-care detection of TB in this patient subset but lacks sensitivity, and its exact role in a diagnostic algorithm for TB in South Africa remains to be clarified.

**Objectives:**

The objective of this study was to better define the patient profile and the TB characteristics associated with a positive urine LAM (LAM+ve) test.

**Method:**

This multicentre retrospective record review examined the clinical, radiological, and laboratory characteristics of hospitalised PLWH receiving urine LAM testing with sputum-scarce and/or negative sputum GeneXpert ^®^ (mycobacterium tuberculosis/resistance to rifampicin [MTB/RIF]) results.

**Results:**

More than a third of patients, 121/342 (35%), were LAM+ve. The positive yield was greater in the sputum-scarce than the sputum-negative group, 66/156 (42%) versus 55/186 (30%), *P* = 0.0141, respectively. Patients who were LAM+ve were more likely to be confused (odds ratio [OR] = 2.2, 95% confidence interval [CI] = 1.2–3.7, *P* = 0.0045), have a higher median heart rate (*P* = 0.0135) and an elevated quick sepsis-related organ failure assessment score (≥ 2), OR = 3.5, 95% CI = 1.6–7.6, *P* = 0.0014. A LAM+ve test was significantly associated with disseminated TB (dTB), *P* < 0.0001, TB-related immune reconstitution inflammatory syndrome (IRIS), *P* = 0.0035, and abdominal TB, *P* < 0.0001. Laboratory predictors of a LAM+ve status included renal dysfunction, *P* = 0.044, severe anaemia, *P* = 0.0116, and an elevated C-reactive protein, *P* = 0.0131. Of the 12 PLWH with disseminated non-TB mycobacteria cultured from the blood and/or bone marrow, *n* = 9 (75%) had a LAM+ve result (OR = 5.8, 95% CI = 1.6–20.8, *P* = 0.0053).

**Conclusion:**

Urine LAM testing of hospitalised PLWH with suspected active TB had significant diagnostic utility in those that were sputum-scarce or sputum-negative. A LAM+ve result was associated with dTB, clinical and laboratory markers of severe illness, and TB-IRIS. Disseminated non-tuberculous mycobacterial infection of hospitalised PLWH may also yield urine LAM+ve results, and mycobacterial cultures must be checked in those non-responsive to conventional TB treatment. Selective use of the LAM test in the critically ill is likely to maximise the diagnostic yield, improve the test’s predictive value, and reduce the time to TB diagnosis and initiation of treatment.

## Introduction

South Africa (SA) has the second highest global incidence of tuberculosis (TB), with an annual estimate of 615 new cases per 100 000 population.^1^ Here, TB is the number one medical cause of death, with more than half of the patients co-infected with HIV.^2^ It is most concerning that in resource-limited settings such as SA, up to 48% of patients co-infected with advanced HIV and TB are undiagnosed ante-mortem.^3^ The accurate diagnosis of TB in persons living with HIV (PLWH) is made difficult by the atypical and often non-specific presentation of disease,^4^ particularly in those with CD4 counts of < 200 cells/µL. This group comprise a substantial proportion (26%) of all SA PLWH^5^ – a group at high risk for HIV-related mortality.

Sputum-based diagnostics, namely the GeneXpert^®^ mycobacterium tuberculosis/resistance to rifampicin (MTB/RIF) (GXP) or the newer Xpert MTB/RIF Ultra assay (Cepheid, Sunnyvale, California, United States of America [USA]), are endorsed by the World Health Organization (WHO)^6^ and recommended where available in local guidelines as a first-line diagnostic^7^ in place of sputum-smear microscopy. Sputum in PLWH with advanced disease may be either test negative,^8^ owing to paucibacillary disease,^4^ or unavailable, based on the lack of pulmonary symptoms or the inability to produce sputum,^9^ thereby limiting the utility of sputum-based diagnostics. Furthermore, the frequency of extrapulmonary TB (EPTB) in this population is high: 40% – 60% of all cases of TB.^4^ A significant proportion of EPTB cases have disseminated TB (dTB), defined as TB in two or more non-contiguous sites,^10^ *Mycobacterium tuberculosis* (MTB) recovered from blood or bone marrow^11^ or miliary TB suspected on chest X-ray (CXR).^12^ If culture-based methods are relied upon, dTB carries high mortality and a risk of significantly delayed diagnosis.^11^

In these populations of PLWH with suspected TB, termed *sputum scarce or sputum negative*, conventional sputum-based diagnostic methods frequently fail, with resultant delayed diagnosis and consequent increased mortality. Resources such as chest radiography or abdominal sonography are often not suggestive of TB^12^ or of inadequate sensitivity and specificity^13^ in this population. Definitive means to establish the diagnosis of TB, such as blood culture, fine needle aspirate, bronchoalveolar lavage or tissue biopsy, are invasive and not always appropriate, and the results of these testing modalities are not always available timeously. Many patients are consequently treated empirically with the associated risk of misdiagnosis.^14^ Alternative diagnostic modalities are urgently needed.

The Alere Determine TB lipoarabinomannan (LAM), a point-of-care (POC) urine-based assay (Supplementary Appendix A), has been recommended by the WHO for a specific subset of PLWH with suspected TB and has the potential to fill this gap. Despite being shown to be less useful in an outpatient setting by Caligaro et al.,^15^ the patient subset with the highest level of evidence includes hospitalised patients with CD4 counts of < 200 cells/µL (previously 100 cells/µL at the time of this study) or those deemed seriously ill by predefined criteria.^16^ The pooled sensitivity and specificity in the recommended population are 45% and 92%, respectively, with the sensitivity inversely proportional to the CD4 count.^17^

The assay detects a form of LAM, a glycolipid component of the mycobacterial outer cell wall. It is one of three major groups of interrelated lipopolysaccharides that are found in all mycobacterial species and released by metabolically active or degrading mycobacteria, and it is detectable *in vitro* in mycobacterial cultures.^18,19^ There is debate as to whether LAM is filtered via the glomerulus in the setting of a possible glomerulopathy from active TB elsewhere or, as the prevailing consensus suggests, whether urine LAM excretion represents haematogenous or other dissemination to the renal parenchyma^20^ or urogenital tract in the context of extrapulmonary TB, or more likely dTB.^21^ This form of EPTB is under-recognised and may be subclinical or suggested by sterile pyuria or unexplained haematuria.^22,23,24^

The relatively poor sensitivity and the ‘restricted’ recommendations that define the test population are perhaps the major contributors towards the under-appreciation and underuse of urine LAM as a diagnostic tool. The fact that at present it is not uniformly available at public sector hospitals in SA contributes to its underuse, and the lack of a large-scale roll-out is possibly linked to the above-mentioned factors. It must be noted, however, that HIV-associated hospital admissions are responsible for up to two-thirds of all public sector hospital admissions,^25^ and up to 25% of the target population (PLWH) are potential recipients of this POC diagnostic.^5^ As demonstrated in the STAMP trial, earlier diagnosis, coupled with the measurable mortality benefit that incorporation of urine LAM testing showed in patients with lower CD4 cell counts, underpins its utility in the SA setting.^26^ It has also been shown to be cost-effective, with a per-unit cost of approximately $3.50 per test.^17,27^

The aim of this study was to identify the parameters associated with a positive urine LAM (LAM+ve) using the Alere Determine TB LAM assay to clarify its optimal usage in an inpatient setting with a view to improving its predictive value and diagnostic yield.

## Objectives

This retrospective study examined the clinical, laboratory, and radiological associations of TB as detected with urine LAM testing of hospitalised PLWH with scarce or test-negative sputum as confirmed on sputum GXP testing. In addition, the study set out to determine what associations (if any) were predictive of a urine LAM+ve result. The utility of this association would be evaluated in a diagnostic algorithm targeting the sputum-scarce or sputum-negative patient population, for whom other diagnostic tools and algorithms often fail.

## Methods

### Design

This was a multicentre retrospective record review of adult patients admitted to the Helen Joseph Hospital and Charlotte Maxeke Academic Hospital in Johannesburg, SA, from 01 January 2017 to 31 December 2017. The eligible study population consisted of hospitalised PLWH with a CD4 count of < 100 cells/µL or who were seriously ill by previously defined criteria^16^ regardless of the CD4 count. These patients were either sputum scarce (unable to produce sputum) or sputum negative using GXP (Xpert; Cepheid, Sunnyvale, California, United States [US]) or on sputum TB culture. Each had a LAM (Alere Determine TB LAM Ag) test performed on a random urine sample.

### Data collection

Records were obtained using a database detailing all patients on whom a urine LAM had been performed in the possession of the department of infectious disease at each hospital. Records were screened for the above eligibility criteria and included if met. Clinical, laboratory and radiological data were retrieved from the same databases, and where incomplete, hospital patient records (if available) were reviewed to complete the record. The data were extracted using manual data collection forms and then captured in spreadsheet form using Microsoft Excel. A total of 361 records were screened, of which 342 met the inclusion criteria (Figure 1). All data obtained were anonymised and given a study number at capture, with the removal of identifiers such as name, date of birth and hospital file number.

**FIGURE 1:**
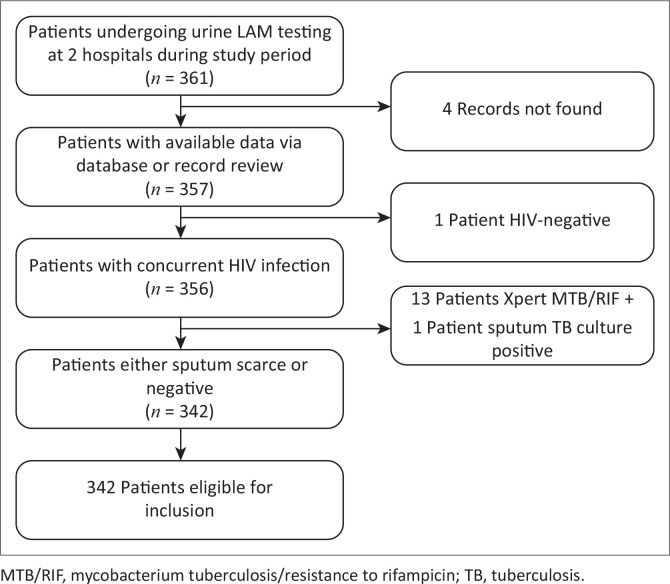
Patient inclusion flow diagram.

### Data parameters

The data extracted included demographic information and clinical data. Clinical data included the vital signs on admission, the presence of danger signs as defined by the WHO^16^ (tachypnoea of more than 30 breaths per minute, fever more than 39 °C, tachycardia greater than 120 beats per minute (bpm), or unable to ambulate), the quick calculated sepsis-related organ failure assessment (qSOFA), calculated^28^ using one point each for confusion, tachypnoea, and systolic BP (SBP) less than 100 mmHg. In addition, features of the WHO TB screening tool,^29^ for the presence of cough, fever, night sweats and loss of weight, were captured. Other parameters included the site of TB if diagnosed (specific sites as defined below), any concurrent diagnoses, the use of antiretroviral treatment (ART) at the time of admission and the presence of virologic failure (VF), defined by the patient being on ART with an HIV viral load (VL) of > 1000 copies/mL.^30^

A diagnosis of pulmonary TB (PTB) was based on the supportive CXR features detailed below, given that sputum diagnostics were an exclusion criterion. The findings deemed suggestive of PTB were recorded in a descriptive format (reticular, nodular, cavitatory, effusion or miliary) or not-PTB (alveolar or normal), having been characterised by the attending clinical team or the primary investigator if the description was not entered but the film was available.^12^

Miliary shadowing on CXR was defined as its own category and considered individually and as a composite part of dTB, as defined previously.

Abdominal TB was defined as TB involving any of the hollow or solid viscera, peritoneum and mesenteric lymph nodes.^10^ A diagnosis in this study was based on either abdominal sonographic features or tissue diagnosis using TB culture or GXP testing from fine-needle aspiration (FNA) or ascitic fluid. Specific abdominal sonographic findings (where performed) suggestive of TB or renal disease (hepatomegaly, splenomegaly, ascites, intra-abdominal lymphadenopathy, splenic hypoechoic lesions^13^ or echogenic/small kidneys) were recorded in descriptive format.

A diagnosis of mycobacteraemia was based on a positive TB blood culture for MTB using the BD BACTEC Myco/F lytic system. The time to positivity was also recorded. Bone marrow TB was diagnosed based on culture of a bone marrow aspirate as above or the confirmation of MTB on a bone marrow trephine (demonstration of acid-fast bacilli and speciation using antigen testing).

A diagnosis of TB lymphadenitis was based on an FNA MTB culture or GXP.

A diagnosis of pleural and pericardial TB was based either on microbiological confirmation of fluid or tissue samples as available or a presumptive diagnosis based on clinical context (exudative effusion with TB symptoms and no alternative diagnosis) or scoring systems such as the Tygerberg score^31^ for echocardiographic pericarditis.

Central nervous system (CNS) TB, including TB meningitis (TBM) or tuberculomas, was based on CSF culture, GXP, clinician gestalt with supportive imaging or CSF findings, as previously described.^32^ Formal scoring systems such as that described elsewhere by Marais et al.,^33^ however, were not incorporated.

A diagnosis of TB-related immune reconstitution inflammatory syndrome (TB-IRIS) was based on the recorded discharge diagnosis and the available clinical history.

Routine laboratory investigations were recorded, including the haemoglobin (Hb), platelet count, white cell count, alanine transferase (ALT), gamma-glutamyl transferase (GGT), alkaline phosphatase (ALP), the presence of an infiltrative or cholestatic liver enzyme pattern (defined by an R-factor of < 2),^34^ (calculated using the ratio of ALT to the upper limit of normal divided by the ratio of ALP to the upper limit of normal), protein, albumin, C-reactive protein (CRP), CD4 count, HIV VL, creatinine and calculated glomerular filtration rate (GFR) using the Modified Diet in Renal Disease formula.^35^

Where available, urine results possibly indicative of renal tract TB (sterile pyuria defined as a urine leukocyte count of > 10 000 cells/µL in the absence of a positive bacterial culture and any associated haematuria) were documented. Similarly, proteinuria (protein:creatinine ratio) indicative of a possible glomerulopathy in patients with a urine LAM+ve was recorded if available.

Patient outcomes, if available, were recorded as inpatient death or survival to hospital discharge.

### Statistics

#### Sample size

A target sample size of 308 was based on data from a previous study,^9^ which found the proportion of sputum-scarce patients with a positive LAM to be 31%, using a 95% confidence interval and Type 1 error value of 0.05. Eligible records yielded a final sample size of 342.

#### Data analysis

The data were analysed using GraphPad Prism, with significance defined as *P* < 0.05 using 95% confidence intervals (CIs). The data were shown to be non-normally distributed using the Kolmogorov–Smirnov test. The results were summarised using descriptive statistics, with categorical variables described using frequencies and proportions, and continuous variables using medians and interquartile ranges. Subgroup univariate analyses were performed for each parameter comparing LAM+ve patients to those with a negative urine LAM (LAM-ve) test. Categorical variables were compared using the chi-squared test (or Fisher’s exact test, where the expected frequencies were < 5). The medians of continuous variables were compared using the Mann–Whitney U-test. Odds ratios (ORs) were calculated with 95% CIs.

### Ethical considerations

Ethical approval for the research was obtained from the University of Witwatersrand Human Research Ethics Committee (ethics clearance certificate number M180815). All retrospective data were anonymised and any personal identifiers removed.

## Results

### Demographics

A total of 342 patients met the study criteria of being admitted to the study hospitals in 2017, being HIV-positive, sputum negative or scarce, and having a urine LAM test performed at the discretion of the managing team in keeping with general WHO recommendations. Of these, 35% (*n* = 121) were LAM+ve. There were no significant differences in patient demographics (Table 1) between the two groups with respect to gender or age.

**TABLE 1:** Patient demographics and outcomes.

Patient demographics and outcomes	*N*	LAM positive			LAM negative			*p*-value
		*n*	%	IQR	*n*	%	IQR	
**Patients**	342	121	35	-	221	65	-	-
**Demographics**								
Median Age, Years (IQR)	-	38	-	32–43	38	-	32–46	0.5411
Male	-	57	47	-	116	52	-	0.3412
Female	-	64	53	-	105	48	-	-
**Outcomes data†**	301	106	35	-	195	65	-	-
Inpatient death	-	21	20	-	25	13	-	0.1074

IQR, interquartile range.

† Limited records with relevant data available.

### Patient outcomes

Regarding the patient outcomes (Table 1), data were available for 301 patients (88%), of whom 46 (15%) died. Higher mortality (not meeting statistical significance) was noted in the LAM+ve group (20% vs 13%, *P* = 0.107). All patients diagnosed with TB were initiated onto treatment in hospital.

### HIV parameters

With regard to HIV-related parameters (Table 2), a significantly larger proportion (80%) in the LAM+ve group had a CD4 of < 50 cells/µL (*P* = 0.0439). Patients in the LAM+ve group were more likely to be on ART at the time of admission, with an OR of 1.7 (95% CI = 1.01–3.0). There was no significant difference in the proportion of patients with combination anti-retroviral therapy (cART) (defined as being in VF on admission with an HIV VL of > 1000 copies/mL) between the groups (64% vs 60%, *P* = 0.6356).

**TABLE 2:** HIV-related parameters.

HIV-related parameters	*N*	LAM positive			LAM negative			*p*-value	Odds ratio	95% CI
		*n*	%	IQR	*n*	%	IQR			
**ART data available**	296†	104	35	-	192	65	-	-	-	-
Currently on ART	-	63	61	-	89	46	-	0.0194*	1.8*	1.1–2.8
**CD4 count**										
Available data	326†	115	35	-	211	65	-	-	-	-
Median, cells/µL (IQR)	-	22	-	7–42	26	-	10–55	0.0944	-	-
< 50 cells/µL	-	92	80	-	147	70	-	0.0439*	1.7*	1.01–3.0
**HIV VL**										
Available data	268†	97	36	-	171	64	-	-	-	-
Median, log copies/mL (IQR)	-	5.2	-	3.5–5.8	5.2	-	3.4–5.9	0.9895	-	-
**Virological failure**										
Available data	134†	56	42	-	72	58	-	-	-	-
VL > 1000 copies/mL on ART	-	36	64	-	47	60	-	0.6356	1.2	0.6–2.3

ART, anti-retroviral therapy; VL, viral load; CD, cluster differentiation; IQR, interquartile range; CI, confidence interval.

* Statistically significant.

† number of records with relevant data available

### Clinical presentation

With regard to presenting symptoms from the WHO TB symptom screen (Table 3), patients with a history of drenching night sweats or loss of weight were significantly more likely to have a positive LAM, 41% versus 25% (*P* = 0.0037) and 54% versus 42% (*P* = 0.039), respectively, with respective ORs of 2 (95% CI = 1.3–3.2) and 1.6 (95% CI = 1.03 to 2.5). Reduced patient mobility was associated with a positive LAM (63% vs 34%, *P* < 0.0001) with an OR of 3.3 (95% CI = 1.9–5.8). Similarly, 41% of patients in the LAM+ve group had abnormal mentation on admission versus 24% of those who were LAM-ve (*P* = 0.0045) with an OR of 2.2 (95% CI = 1.3–3.7).

**TABLE 3:** Clinical features.

Clinical features	*N*	LAM positive			LAM negative			*p*-value	Odds ratio	95% CI
		*n*	%	IQR	*n*	%	IQR			
**TB symptoms**	342	121	-	-	221	-	-	-	-	-
Cough	-	56	46	-	106	48	-	0.7657	0.9	0.6–1.5
Fever	-	39	32	-	56	25	-	0.1736	1.4	0.9–2.3
Night sweats	-	49	41	-	56	25	-	0.0037*	2.0	1.3–3.2
Weight loss	-	65	54	-	93	42	-	0.039*	1.6	1.03–2.5
**Mobility**	223†	80	-	-	143	-	-	-	-	-
Immobile	-	50	63	-	48	34	-	< 0.0001*	3.3	1.9–5.4
**Mentation**	260†	95	-	-	165	-	-	-	-	-
Confused	-	39	41	-	40	24	-	0.0045*	2.2	1.2–3.7
**Vital signs**										
**MAP**	338†	121	-	-	217	-	-	-	-	-
Median, mmHg (IQR)	-	83	-	73–91	86	-	75–95	0.0398*	-	-
MAP < 65	-	10	8	-	8	4	-	0.0723	2.4	0.9–6
**HR**	338†	121	-	-	217	-	-	-	-	-
Median, bpm (IQR)	-	110	-	96–123	105	-	90–115	0.0135*	-	-
HR > 110	-	57	47	-	71	33	-	0.0089*	1.8	1.2–2.9
**Temperature,**	328†	119	-	-	209	-	-	-	-	-
Median, °C (IQR)	-	37	-	36.5–37	37	-	36–37	0.2335	-	-
**RR**	225†	80	-	-	145	-	-	-	-	-
Median, breaths per minute (IQR)	-	20	-	18–24	20	-	18–22	0.0823	-	-
**qS0FA score**	225†	80	-	-	145	-	-	-	-	-
0	89	26	29	-	63	71	-	0.0075*	-	-
1	107	36	34	-	71	66	-	-	-	-
2	27	16	59	-	11	41	-	-	-	-
3	2	2	100	-	0	0	-	-	-	-
qS0FA ≥ 2	29	18	23	-	11	8	-	0.0014*	3.5	1.6–7.6

LAM, lipoarabinomannan; MAP, mean arterial pressure; HR, heart rate; RR, respiratory rate; qS0FA, quick sequential organ failure assessment; bpm, beats per minute; IQR, interquartile range; CI, confidence interval.

* Statistically significant.

† Number of records with relevant data available.

With regard to vital signs on admission, patients in the LAM+ve group had a significantly lower median mean arterial pressure of 83 mmHg (interquartile range [IQR] = 73–91) versus 86 mmHg (IQR = 75–95), a higher median heart rate (HR) of 110 bpm (IQR = 96–123) versus 105 bpm (IQR = 90–115, *P* = 0.0135) and were more likely to have an HR greater than 110 bpm (OR = 1.8, CI = 1.2–2.9). Of note was the proportion of patients in the LAM+ve group who directly correlated with an increasing qSOFA score, namely 29%, 34%, 59% and 100%, respectively, for qSOFA scores equal to 0, 1, 2 and 3 (*P* = 0.0075). In addition, a qSOFA score of greater than or equal to 2 had an OR of 3.5 (95% CI = 1.6–7.6) for being LAM+ve.

### Tuberculosis diagnosis

Of those diagnosed with TB (*n* = 168), 72% (*n* = 121) were LAM+ve (Table 4). Of those who were LAM+ve, the urine LAM test was the only microbiological modality of TB confirmation that was positive in more than half (52%).

**TABLE 4:** Tuberculosis diagnosis.

TB diagnosis	*N*	LAM positive (*n* = 121)			LAM negative (*n* = 121)			*p*-value	Odds ratio	95% CI
		*n*	%	IQR	*n*	%	IQR			
**Sputum results**										
Sputum scarce	156	66	42	-	90	58	-	0.0141*	1.7	1.3–2.7*
Sputum negative	186	55	30	-	131	70	-	-	-	-
**TB diagnosis (all sites)**	168	121	72	-	47	28	-	-	-	-
Microbiology (PCR/Culture) positive	48	34	71	-	14	29	-	0.8279	0.9	0.4–1.9
Microbiology (PCR/Culture) negative	120	87	73	-	33	27	-	-	-	-
LAM sole diagnostic modality†	-	87	52	-	-	-	-	-	-	-
**Site involved**										
Disseminated disease‡	52	43	83	-	9	17	-	< 0.0001*	13.0	6.0–27.8
Miliary^21^	10	7	70	-	3	30	-	0.0376*	4.5	1.2–16.1
Bone Marrow	7	7	100	-	0	0	-	0.0006*	∞	3.4–∞
Mycobacteraemia	33	27	82	-	6	18	-	< 0.0001*	8.4	3.3–20.3
Median time to positivity, days	-	21	-	16–28	22	-	15–27	0.7759	-	-
Pulmonary§	28	22	79	-	6	21	-	< 0.0001*	8.0	3.1–19.2
Abdominal	55	40	73	-	15	27	-	< 0.0001*	6.8	3.5–12.5
Lymph node	10	6	60	-	4	40	-	0.1753	2.8	0.7–9.0
CNS	18	7	39	-	11	61	-	0.7491	1.7	0.5–3.1
Pleural	5	1	20	-	4	80	-	0.6597	0.5	0.0–2.8
Pericardial	1	0	0	-	1	100	-	> 0.9999	0	0.0–16.4
Spinal	1	1	100	-	0	0	-	0.3538	∞	0.2–∞
IRIS	8	7	88	-	1	12	-	0.0035*	13.5	2.2–152.8
No anatomical localisation	42	42	100	-	0	0	-	-	-	-

LAM, lipoarabinomannan; PCR, polymerase chain reaction; CNS, central nervous system; IQR, interquartile range; IRIS, immune reconstitution inflammatory syndrome; CI, confidence interval.

* Statistically significant.

† Of those with final TB diagnosis, urine LAM was the only microbiological diagnostic method found to be positive;

‡ TB in two or more non-contiguous sites, recovery of MTB from blood or bone marrow, or radiological evidence of miliary TB;

§ Diagnosis based on chest radiography.

Grouping patients as either sputum scarce or sputum negative, they were significantly more likely to be LAM+ve in the sputum-scarce group (42% vs 30%, *P* = 0.0141), perhaps reflecting the negative predictive value of a negative GXP result.

Of those patients diagnosed with dTB (as previously defined), 83% were LAM+ve (*P* < 0.0001) with an OR of 13 (95% CI = 6.0–27.8). Subsets of those with dTB included those with CXR features of miliary TB (*n* = 10), in whom 70% were LAM positive (*P* = 0.0376). There was also a significant correlation with mycobacteraemia, with an OR of 8.4 (95% CI = 3.4–20.4) for a positive LAM.

Regarding other sites of involvement (often more than one), 73% (*n* = 40) of patients with abdominal TB had a positive LAM (*P* < 0.0001). Bone marrow involvement was predictive of a positive LAM in 100% (n = 7) of patients (P = 0.0006). Similarly, a diagnosis of TB-IRIS at any site had an OR of 13.5 (95% CI = 2.2–152). Although the numbers are small, *n* = 7 (88%) of patients with TB-IRIS had a positive LAM (*P* = 0.0035).

No significant correlation was demonstrated with TB involvement of the CNS (tuberculomas or TBM), lymphadenitis, pleura, pericardium or spine (Pott’s disease), although sample sizes were small for all these subanalyses and thus did not reach significance.

### Laboratory investigations

With regard to haematological parameters (Table 5), LAM+ve patients had a significantly lower median haemoglobin of 9.1 g/dL (IQR = 7–11) versus 10.6 g/dL (IQR = 8.5–15, *P* < 0.0001). In addition, the presence of severe anaemia as per the WHO definition^36^ (Hb < 8 g/dL for men and < 7 g/dL for women) had an OR of 2 (95% CI = 1.2–3.5) for a positive LAM.

**TABLE 5:** Laboratory results.

Laboratory markers	*N*	LAM positive			LAM negative			*p*-value	Odds ratio	95% CI
		*n*	%	IQR	*n*	%	IQR			
**WCC (×10⁹ cells/L)**	339¶	119	-	-	221	-	-	-	-	-
Median (IQR)	-	6	-	3.9–9.1	5.1	-	3.2–8	0.1263	-	-
> 11	-	20	17	-	26	12	-	0.1948	1.5	0.8–2.8
< 4	-	31	26	-	79	36	-	0.0683	0.6	0.4–1
**Hb (g/dL)**	341¶	120	-	-	221	-	-	-	-	-
Median (IQR)	-	9.1	-	7–11	10.6	-	8.5–12	< 0.0001*	-	-
Severe anaemia†	-	30	25	-	31	14	-	0.0116*	2	1.2–3.5
**Platelets (×10⁹ cells/L)**	340¶	119	-	-	221	-	-	-	-	-
Median	-	240	-	141–347	263	-	177–336	0.3306	-	-
< 100	-	14	12	-	21	10	-	0.5126	1.3	0.6–2.6
> 450	-	10	8	-	20	9	-	0.8411	0.9	0.4-2
**GFR (mL/min/1.73m²)**	340¶	119	-	-	221	-	-	-	-	-
Median	-	73	-	44–103	85	-	56–109	0.068	-	-
< 60	-	49	41	-	67	30	-	0.044*	1.6	1–2.5
**Liver function tests (g/dL or IU/L)**										
Available data	334¶	119	36	-	215	64	-	-	-	-
Median Albumin	-	24	-	19–47	25	-	20–43	0.474	-	-
< 30	-	94	79	-	150	70	-	0.0688	1.6	1–2.7
Available data	328¶	117	-	-	211	-	-	-	-	-
Median GGT	-	85	-	45–162	68	-	40–133	0.0805	-	-
GGT > ULN	-	72	62	-	105	50	-	0.0404*	1.6	1–2.5
Available data	327¶	115	-	-	212	-	-	-	-	-
Median ALT	-	36	-	20–66	17	-	17–52	0.0174*	-	-
ALT > ULN	-	51	44	-	75	35	-	0.1115	1.5	0.9–2.3
Infiltrative LFT‡	-	39	34	-	61	19	-	0.3355	1.3	0.8–2.1
**CRP (mg/L)**	317¶	115	-		202	-		-	-	-
Median	-	125	-	54–200	91		23–157	0.0131*	-	-
> 100	-	72	63	-	94	47	-	0.0059*	1.9	1.2–3.1
**Urinalysis**										
Available data	103¶	47	-	-	56	-	-	-	-	-
Sterile pyuria§	-	15	32	-	20	36	-	0.6851	0.8	0.4–1.8
Available data	59¶	31	-	-	28	-	-	-	-	-
Median PCR, g/μmol (IQR)	-	0.157	-	0.115–0.379	138	-	0.057–0.261	0.1491	-	-
Nephrotic proteinuria (> 3.5g/μmol)	-	8	26	-	2	7	-	0.0838	4.5	0.9–22.4

LAM, lipoarabinomannan; WCC, white cell count; Hb, haemaglobin; GFR, glomerular filtration rate; GGT, gamma-gutamyl transferase; PCR, protein:creatinine ratio; ULN, upper limit normal; LFT, liver function tests; ALT, alanine-amino transferase; CRP, C-reactive protein; IQR, interquartile range; CI, confidence interval.

* Statistically significant.

† Hb < 8g/dL for men and < 7g/dL for women;

‡ Defined by R factor < 2 calculated with the formula (ALT × ALP ULN) ÷ (ALP × ALT ULN);

§ > 10000 cells/μL in the absence of bacterial culture;

¶ Number of records with relevant data available.

With regard to renal function, a significantly higher proportion of LAM+ve patients had abnormal renal function, defined by a GFR of < 60 mL/min/1.73 m^2^, 41% versus 30% (*P* = 0.044) with a OR of 1.6 (95% CI = 1.0–2.5). However, with respect to the available urine parameters, there was no difference in the presence of sterile pyuria (*P* = 0.6851) between the two groups. There was a higher proportion of patients in the LAM-positive category with nephrotic range proteinuria (26% vs 7%), trending towards statistical significance (*P* = 0.0838).

Regarding hepatic enzyme levels, LAM+ve patients were more likely to have a GGT above the upper limit of normal, defined as greater than 68 U/L, with an OR of 1.6 (95% CI = 1.02–2.5), with 62% in the LAM-positive group having an abnormal GGT versus 50% in the LAM-negative group (*P* = 0.0404). There was no significant difference between the two groups with regard to the presence of an infiltrative liver function test (LFT), defined by an R-factor of < 2 as defined above, 34% versus 29% (*P* = 0.3355).

With regard to inflammatory markers, patients who were LAM+ve had a significantly higher median CRP of 125 mg/L (IQR = 54–354) versus 91 mg/L (IQR = 23–157), with *P* = 0.0131. The presence of a CRP of >100 mg/L had an OR of 1.9 (95% CI = 1.2–3.1) for a positive LAM.

### Radiology

In classifying specific CXR findings, the presence of nodular and miliary infiltrates was associated with a positive LAM with ORs of 2.5 (95% CI = 1.2–5.3) and 4.3 (95% CI = 1.2–15.6), respectively (Table 6). Of those patients with a miliary pattern on CXR (*n* = 10), 70% had a positive LAM (*P* = 0.0394).

**TABLE 6:** Radiology.

Radiology	*N*	LAM positive		LAM negative		*p*-value	Odds ratio	95% CI
		*n*	%	*n*	%			
**Chest radiography†**	307	111	-	196	-	-	-	-
Normal	104	35	34	69	66	0.5136	0.8	0.5–1.4
Interstitial infiltrate	75	25	33	50	67	0.5583	0.8	0.5–1.5
Nodular infiltrate	30	17	57	13	43	0.0138*	2.5	1.2–5.3
Alveolar infiltrate	18	0	0	18	100	0.0005*	0	0–0.3
Consolidation	39	17	44	22	56	0.3011	1.4	0.7–2.8
Cavitation	17	6	35	11	65	0.9999	1	0.4–2.9
Effusion	14	2	14	12	86	0.094	0.3	0.1–1.1
Hilar lymphadenopathy	21	9	43	12	57	0.4921	1.4	0.6–3.1
Miliary pattern	10	7	70	3	30	0.0394*	4.3	1.2–15.6
**Abdominal sonography‡**	142	56	-	86	-	-	-	-
Normal	46	15	33	31	67	0.2491	0.6	0.3–1.4
Ascites	21	9	43	12	57	0.7282	1.2	0.5–2.9
Splenic microabscesses	21	19	66	10	34	0.0013*	3.9	1.7–9.3
Splenomegaly	14	7	50	7	50	0.3943	1.6	0.6–4.4
Lymphadenopathy	29	18	62	11	38	0.0052*	3.2	1.4–7.3
Hepatomegaly	24	12	50	12	50	0.2454	1.7	0.7–3.9
Hepatic lesion/s	6	2	33	4	67	0.9999	0.8	0.1–3.4
Echogenic kidneys	15	5	33	10	67	0.7818	0.7	0.3–2.2

LAM, lipoarabinomannan; IQR, interquartile range; CI, confidence interval.

* Statistically significant.

† As characterised by clinician or researcher;

‡ As per sonography report.

Regarding abdominal sonography for features of abdominal TB or renal disease, a positive LAM was associated with the presence of splenic micro-abscesses with an OR of 3.9 (95% CI = 1.7–9.3) and intra-abdominal lymphadenopathy with an OR of 3.2 (95% CI = 1.4–7.3).

### Concurrent diagnoses

Selected HIV-associated diagnoses were considered to identify possible screening populations with the potential for a significant yield (Table 7). No significantly increased frequency of LAM positivity was noted in patients with an isolated positive serum cryptococcal latex agglutination test, cryptococcal meningitis (CCM) or community-acquired pneumonia. A negative association was noted in patients with *Pneumocystis jirovecii* pneumonia (PJP), with 11% patients with PJP having a positive LAM versus 89% LAM negative (*P* < 0.0001). Given that lymphoproliferative disorders are often misdiagnosed as TB,^14^ it was significant that in patients with a final diagnosis of lymphoma (*n* = 8), none had a positive LAM (*P* = 0.0053).

**TABLE 7:** Concurrent diagnoses.

Selected concurrent diagnoses	*N*	LAM positive (*n* = 121)		LAM negative (*n* = 221)		*p*-value	Odds ratio	95% CI
		*n*	%	*n*	%			
**Cryptococcal disease**								
CLAT†	16	8	50	8	50	0.2103	1.9	0.7–5.1
CCM	52	14	27	38	73	0.166	0.6	0.3–1.2
CAP	46	11	24	35	76	0.0804	0.5	0.3–1.1
PJP	54	6	11	48	89	< 0.0001*	0.2	0.1–0.4
**NTM (disseminated)**‡	12	9	75	3	25	0.0053*	5.8	1.6–20.3
Mycobacterium avium complex	11	8	73	3	27	-	-	-
*M. kansasii*	1	1	100	0	0	-	-	-
Lymphoma	13	0	0	13	100	0.0053*	0	0–0.5
AKI	70	30	43	40	57	0.1424	1.5	0.9–2.5

CCM, cryptococcal meningitis; CAP, community acquired pneumonia; PJP, *pneumocystis jirovecci* pneumonia; NTM, non-tuberculous mycobacteria; AKI, acute kidney injury.

* Statistically significant.

† Isolated cryptococcal antigen detection on serum without meningitis;

‡ Isolated from blood culture or bone marrow.

Also of significance was that of the 12 patients with disseminated non-tuberculous mycobacterial (NTM) infection, *n* = 9 (75%) had a positive LAM (*P* = 0.0053) with an OR of 5.8 (95% CI = 1.6–20.2). These included eight patients with disseminated *Mycobacterium avium* complex (MAC) and one patient with disseminated *Mycobacterium kansasii.*

## Discussion

The results of this research support urine LAM as a clinically useful diagnostic modality in this study population. Several associations were identified. Over one-third (*n* = 121, 35%) of patients in this group of sputum-scarce or sputum-GXP-negative hospitalised PLWH with a CD4 count of < 100 cells/µL, or deemed to be severely ill regardless of the CD4 count, had a positive urine LAM. Of the 168 patients diagnosed with TB in the study population, being either sputum negative or sputum scarce, a urine LAM was the only microbiological confirmatory modality of TB in half (*n* = 87, 52%). Indeed, where sputum-based diagnostics are negative or not feasible, as in many sick PLWH, alternate diagnostics that are easy to implement, affordable and provide rapid results can be life-saving. A higher proportion of those who were sputum scarce (42%) were LAM+ve compared to the sputum-negative cohort (30%). This is in keeping with the negative predictive value of a sputum GXP. In keeping with the findings of other studies, urine LAM was positive in the majority (71%) of those with microbiologically confirmed TB.^37^

As demonstrated in other studies,^37,38,39^ the yield (and sensitivity) of urine LAM testing is inversely proportional to the CD4 count: a higher yield of LAM+ve results was obtained in our patients with CD4 counts of < 50 cells/µL (80% vs 20%) with an OR of 1.7. While HIV VL or the presence of VF were not significantly different between those who were LAM+ve or LAM-ve, those in the positive group were more likely to also be on ART – possibly implicating TB-IRIS, particularly in light of the high OR (13.5) and level of LAM positivity (88%) in this group.

In this study, patients who were LAM positive had a higher overall mortality, namely 20% versus 13% for LAM-negative patients. The mortality data were similar in the TB group. Neither met statistical significance (*P* = 0.107), a finding in keeping with other studies.^38^ This is of interest, given that previous research by Peter et al.^40^ and the recent STAMP trial^26^ showed a mortality reduction by incorporating urine LAM into the diagnostic algorithm for TB.

Interesting clinical features with a positive predictive value for a positive urine LAM included patients with reduced mobility and confusion, a unique subset in whom sputum-based diagnostics would be difficult to obtain, once again highlighting the usefulness of the urine LAM test. Other notable clinical features associated with a positive LAM included a significantly lower median blood pressure and a higher median HR, suggesting an overall sicker patient group. A qSOFA score^28^ showed a significant correlation with proportional increases in LAM yield relative to a higher score for a given patient. This, together with the higher mortality, indicates a seriously ill patient population in whom diagnostic urgency and the early initiation of treatment are imperatives.

With regard to the nature of the underlying TB diagnosis, several results from this study are pertinent. Firstly, there was a significant association of urine LAM with more disseminated forms of TB, namely mycobacteraemia, TB infection of the bone marrow, miliary TB and TB involving two or more non-contiguous sites. This group had a cumulative OR for a positive LAM of 13.5. Secondly, 82% of those with mycobacteraemia had a positive LAM. The importance of this is amplified when the 25 min it takes to run a LAM is contrasted with the median blood culture time to positivity of 21 days. This was also in agreement with similar findings from other studies.^37,41^ Thirdly, LAM is useful in the interpretation of abdominal sonographic features of TB, which in isolation are only of modest sensitivity (63%) and specificity (68%).^13^ Finally, it should be noted that in 33% of LAM+ve patients *no* obvious site of disease was identified with available clinical and laboratory support. Thus LAM provides screening utility where other diagnostic modalities currently fail.

The analysis of laboratory parameters associated with a positive urine LAM included the presence of severe anaemia (OR = 2.0) and a GFR of less than 60 mL/min/1.73m^2^ (OR = 1.6). Regarding liver function testing, which is often used as a surrogate for the presence of abdominal TB in high endemic areas, urine LAM positivity was significantly associated with an abnormal GGT, and a higher median ALT, but no significant association was found with an infiltrative/cholestatic LFT picture defined by an R-factor (as defined previously) of < 2.0.^34^ Lastly, a positive LAM correlated with an elevated median CRP, with values of > 100 mg/L having an OR of 1.9.

Various urinary parameters were available for a subset of patients, but no association was found between patients with a positive LAM and sterile pyuria, a marker of possible renal TB. An increased proportion of patients with nephrotic range proteinuria in the LAM-positive group approached statistical significance (*P* = 0.0838), possibly representing an increase in the glomerular filtration of LAM from active sites of disease elsewhere in patients with underlying HIV-associated nephropathy (HIVAN). Alternatively, it may be coincidentally related to the high prevalence of HIVAN in this population with advanced HIV and low CD4 counts.^42^

Patients with abdominal TB and intra-abdominal lymphadenopathy or splenic micro-abscesses on sonar were more likely to have a positive urine LAM. Given the non-specific sonographic features and the large variety of clinical possibilities – TB, lymphoma, fungal infection, NTM infection, sarcoid, bartonella^43^ – the urine LAM, a simple test that confirms dTB, is readily justifiable in the situation.

There exists debate in the literature as to the relevance of the finding of urine LAM in patients with confirmed disseminated (diagnosed on blood culture) NTM disease, namely whether it represents a confounder of the MTB result and is related to the NTM infection directly,^44^ or given the high prevalence of TB in this population, perhaps the presence of concurrent undiagnosed TB.^45^ In this study, of the 12 patients with confirmed disseminated NTM infections, 75% (*n* = 9) had a positive urine LAM. Despite the small sample this was in keeping with the findings of Nel et al.^44^ Of interest in respect to this debate was that in one of these patients, concurrent disseminated TB was confirmed on a blood culture. The balance though does seem to suggest that disseminated NTM infection can cause a positive urine LAM, and this should be kept in mind when interpreting a urine LAM result. Although not statistically significant, over 25% of patients with CCM had a positive urine LAM (*n* = 14). While not common, concurrent TB (including TBM) and cryptococcal disease have been reported in a number of at-risk populations.^46,47^ Further research is indicated in this regard as to whether screening using urine LAM may have a role in this patient subset.

With its clearly demonstrated utility, it does need to be emphasised that while in resource-constrained settings it may be the only modality of diagnosis, and while it may improve the time to diagnosis, other means to confirm mycobacterial disease (PCR or culture-based methods) should still be sought where possible so as not to fail to identify drug resistance and differentiate NTM from TB infections.

## Limitations

The limitations of the study include the retrospective nature of the data obtained with the associated risk of information and misclassification bias. In classifying patients as sputum negative, the quality of the sputum was not known, which may have resulted in the erroneous inclusion of sputum-positive patients. The diagnosis of TB-IRIS was based on the discharge diagnosis as per the clinical records and meeting of the formal case criteria^48^ was not always confirmed. Similarly, cases of VF were defined based on a detectable HIV VL in a patient already on ART, but the duration of ART use was not available. Several records were incomplete, and thus only the available data were analysed. Cases defined as having abdominal TB based on sonography alone may have been misclassified, given the differentials of sonographic features such as splenic microabscesses.^43^ It has been reported with an earlier generation LAM enzyme-linked immunosorbent assay that *Candida* species may cause a false-positive LAM.^49^ This cannot be excluded as a confounder on untested specimens, but no urine samples in this study that were cultured and also tested positive for urine LAM demonstrated *Candida*. Small samples in several subanalyses limited the statistical analysis.

## Conclusion

Urine LAM is a simple, easy-to-perform POC test that takes only 25 min to establish a result. While further research is indicated to determine the optimal implementation strategies, this study demonstrates that it had significant utility in hospitalised PLWH with CD4 counts of less than 100 cells/µL or in the seriously ill, who were either sputum scarce or sputum negative, with a higher yield in the former. In this cohort of patients, the definitive diagnosis of TB is both elusive and often delayed, with dire consequences. A positive LAM result predicted a seriously ill subset of patients with a treatable cause yet with a significant risk of mortality, in whom a mortality benefit of including the test in a diagnostic algorithm has been previously demonstrated. A positive urine LAM result was also strongly associated with several disseminated and extrapulmonary forms of the disease, specifically mycobacteraemia, bone marrow involvement and abdominal TB, of which diagnosis in resource-limited settings is often difficult or delayed. It was also helpful and accurate in establishing the diagnosis of TB-IRIS where suspected. Several laboratory associations were established, including the presence of severe anaemia, renal dysfunction and abnormalities on liver function testing, and an increased yield in those patients who were immobile or confused, with these possibly guiding its rational usage in a diagnostic algorithm (suggested example in Appendix B), with potential to improve its positive and negative predictive values. Possible false-positive results may be encountered in patients with disseminated NTM infections, including MAC and *Mycobacterium kansasii*. Nevertheless, the urine LAM test is a definite advance in the early confirmation of life-threatening infection with MTB in PLWH with advanced immunosuppression.

## Figures and Tables

**FIGURE 1-A1:**
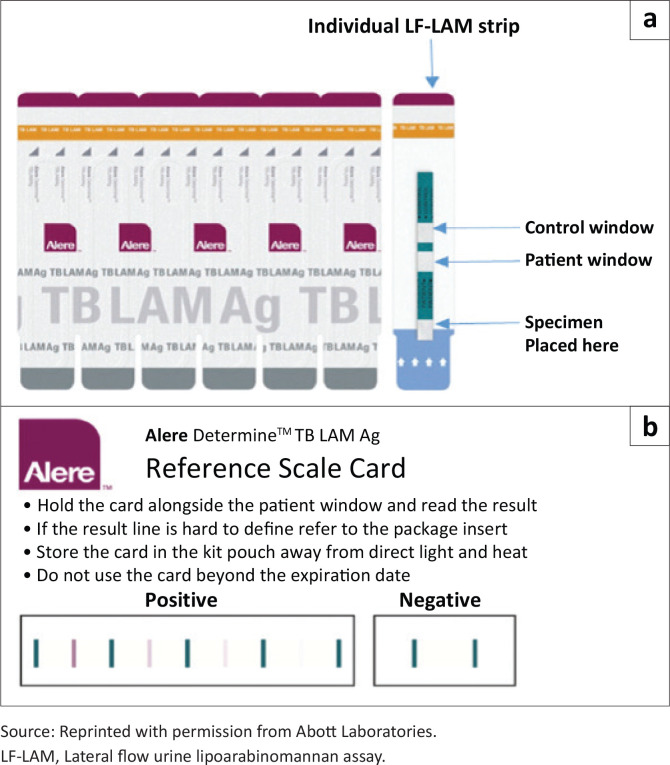
Alere Determine™ TB LAM Ag: (a) assay and (b) reference card.

**FIGURE 1-A2:**
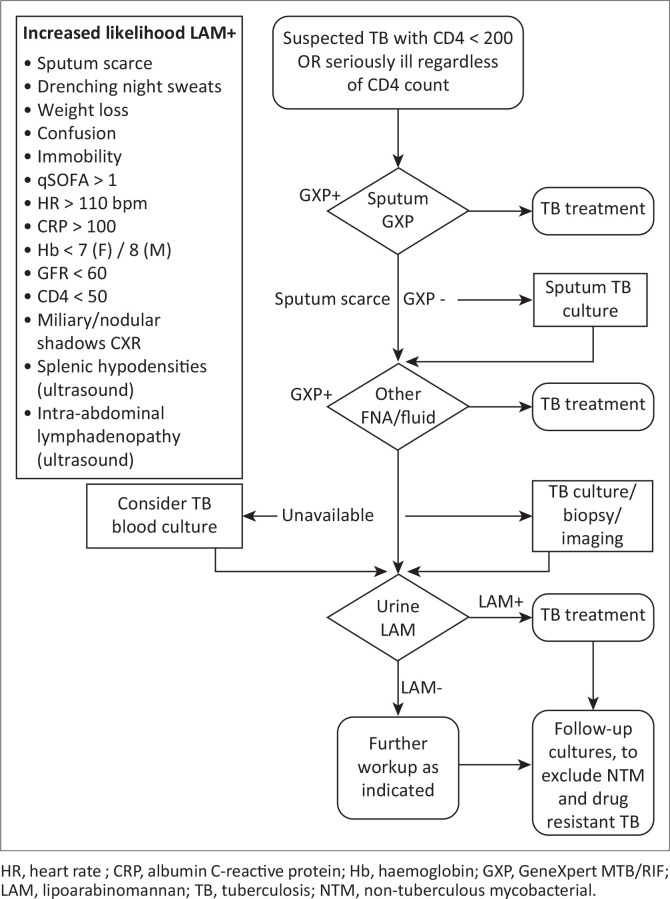
Suggested algorithm for usage of urine LAM in hospitalised patients.

## Appendix A

**Alere TB LAM Ag**

The Alere Determine TB LAM Ag (Abott Laboratories, Chicago, Illinois, US) shown in Figure 2A is a point-of-care lateral flow-based assay. A sample of 65 µL of urine is placed on the testing strip using an enclosed pipette, which flows up the testing strip. This attaches colloidal gold-labelled antibodies to LAM that are captured by immobilised LAM antibodies further along the test strip. These form a visual band, which is then compared to a reference card (Figure 2B) for interpretation, dependent on the band visibility and intensity (positive being considered grades 1–4, as illustrated). The result is read at 25 min.
